# Feasibility of hybrid Ivor-Lewis oesophagectomy after sleeve gastrectomy

**DOI:** 10.1093/icvts/ivab288

**Published:** 2021-10-18

**Authors:** Guy Pines, Harbi Khalayleh, Ibrahim Mashni, Ilan Bar

**Affiliations:** 1 Faculty of Medicine, Hebrew University of Jerusalem, Jerusalem, Israel; 2 Department of Thoracic Surgery, Kaplan Medical Center, Rehovot, Israel; 3 Department of Surgery, Kaplan Medical Center, Rehovot, Israel

**Keywords:** Oesophagectomy, Sleeve gastrectomy, Oesophageal cancer

## Abstract

Oesophageal resection is a challenging procedure, and it is more so in patients who have undergone bariatric procedures, especially after laparoscopic sleeve gastrectomy. We present the case of a patient with a history of an laparoscopic sleeve gastrectomy who underwent a hybrid Ivor-Lewis oesophagectomy in which the sleeve was successfully used to reconstruct the upper gastrointestinal tract.

## CASE HISTORY

A 56-year-old man presented with a 6-cm Siewert I poorly differentiated oesophageal adenocarcinoma, staged as cT3N2M0. He lost 20% of his body weight, which was 88 kg on admission. His medical history was significant for a laparoscopic sleeve gastrectomy (LSG) 7 years ago, severe symptomatic diverticulosis of the descending and sigmoid colon, and a giant sigmoid diverticulum.

**Figure 1:**
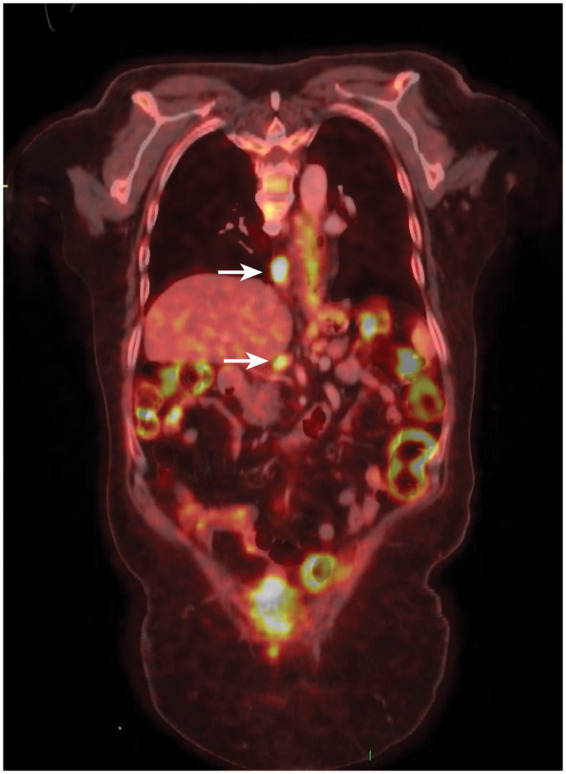
A restaging positron emission/computed tomography scan showing 2 enlarged paraoesophageal and coeliac lymph nodes (white arrows).

Following neoadjuvant therapy (FLOT protocol—Fluorouracil, Folinic acid, Oxaliplatin, Docetaxel), partial downstaging was achieved. The oesophageal mass could not be detected, and only 2 lymph nodes showed decreased, yet persistent, fluorodeoxyglucose (FDG) uptake (Fig. 1). The operative plan was to attempt to salvage the gastric sleeve if possible, or to perform a jejunal interposition if this failed, thus avoiding the diseased colon.

The patient underwent a hybrid Ivor-Lewis oesophagectomy (Video 1). We used a 5-port approach. After freeing the gastric sleeve from previous adhesions, the left gastric vessels were divided. The length of the stomach seemed appropriate for reconstruction. The stomach was divided 4–5 cm distal to the gastro-oesophageal junction and sent for frozen section analysis, the results of which were negative. As the stomach, now based on the right gastric vessels, seemed well perfused on gross inspection, a decision was made to use it as a conduit. A feeding jejunostomy was fashioned, and dissection of the distal oesophagus was carried out via 4-port right video-assisted thoracoscopy. The oesophagus was circumferentially dissected up to the level of the azygos vein, and the gastric conduit was brought into the thoracic cavity. The stomach safely reached above the tumour, ensuring clear proximal margins. The oesophagus was transected 3 cm distal to the azygos vein. A gastrotomy was performed, but the shaft of the circular stapler could not be safely delivered. A mini-thoracotomy was performed, and an end-to-side anastomosis was performed using a 25-mm EEA OrVil stapler (Medtronic, Dublin, Ireland). The patient had an uneventful postoperative course and was discharged on postoperative day 9.

The final pathological report was consistent with ypT2N3(11/16)M0 adenocarcinoma because only a few tumour cells were identified in the muscularis. Lower mediastinal and coeliac nodes were positive. All upper mediastinal level 7 and 8 nodes were negative.

## DISCUSSION

Patients who have had a sleeve gastrectomy pose a challenge during oesophagectomy, because the greater curvature is resected during the index procedure. Reconstruction options include the colon and the jejunum. Only 2 cases have been reported in which the gastric sleeve was used for reconstruction. Pallabazzer *et al.* [1] reported a case of a 53-year-old woman who developed oesophageal adenocarcinoma 7 years after an LSG. She underwent upfront surgery. The conduit was created by sequential firing of a linear stapler starting alongside the lesser curvature, with the creation of a new sleeve of about 5 cm width. Gastric vascularization was evaluated by fluorescence after injection of indocyanine green. The anastomosis was created at the level of the azygos vein.

The second case was that of a 61-year-old man [2], 4 years after laparoscopic biliopancreatic diversion with a duodenal switch. The left gastric artery was clamped, and the biliopancreatic diversion with a duodenal switch was reversed. The gastric vascularization evaluation was made on gross inspection and endoscopic assessment. A transhiatal resection and a cervical anastomosis were performed.

The greater curvature of the stomach provides ample length for reconstruction. The length of the sleeve conduit is less predictable. In the case presented here, no Kocherization was performed to minimize injury risk to the conduit vascular supply, which reached the level of the carina. Because this was a Siewert I tumour, an intraoperative evaluation was performed to assess the conduit length. At least 10–12 cm of conduit could reach the hiatus, ensuring adequate proximal resection margins. Among patients who underwent LSG, the stomach could safely be used for reconstruction in tumours of the distal oesophagus and the gastro-oesophageal junction. It may not be applicable in patients with mid-oesophagus tumours, because a standard Ivor-Lewis oesophagectomy with a high mediastinal anastomosis may not always be possible, as in this case. Another limitation to this approach, even when adequate margins are achieved, is a somewhat limited lymphadenectomy, especially in the upper mediastinum and along the lesser curvature.

The other concern among patients who underwent an LSG is conduit perfusion. This, however, might not be an issue, mainly due to the interval from the bariatric procedure, which may provide ischaemic conditioning to the stomach. Laparoscopic ischaemic conditioning with division of the left gastric vessels prior to oesophagectomy has been proposed to allow neovascularization of the conduit [3] and decrease the risk of anastomotic complications [4]. Better results may be expected with a prolonged preconditioning period [5]. Among LSG patients, the short gastric and the greater curvature vessels have been divided. After several years have, ischaemic preconditioning might contribute to a lower anastomotic leak risk.

## CONCLUSIONS

Among patients who underwent an LSG, the stomach could safely be used for reconstruction in tumours of the distal oesophagus and the gastro-oesophageal junction. Previous bariatric surgery might contribute to a lower risk of an anastomotic leak because of the ischaemic preconditioning.

### Disclosures

The authors have no disclosures or conflict of interests.

## Reviewer information

Interactive CardioVascular and Thoracic Surgery thanks Hasan Fevzi Batirel, Olli Helminen and the other, anonymous reviewer(s) for their contribution to the peer review process of this article.
